# Occupational radiation exposure assessment in tin smelting: an integrated evaluation of external and internal doses from NORM in Indonesia

**DOI:** 10.3389/fpubh.2026.1808363

**Published:** 2026-05-04

**Authors:** Ilsa Rosianna, Radhia Pradana, Eka Djatnika Nugraha, Wahyudi Wahyudi, Muhammad Muhyidin Farid, Sulaksana Permana, Adi G. Muhammad, Muflihatul Muniroh, Hasnawati Amqam, Chutima Kranrod, Donovan Anderson, Hirofumi Tazoe, Shinji Tokonami, Naofumi Akata

**Affiliations:** 1 Department of Radiation Science, Hirosaki University Graduate School of Health Sciences, Aomori, Japan; 2 Research Center for Nuclear Fuel Cycle and Radioactive Waste Technology, Research Organization for Nuclear Energy (ORTN), National Research and Innovation Agency (BRIN), South Tangerang, Indonesia; 3 Research Center for Safety, Metrology, and Nuclear Quality Technology, Research Organization for Nuclear Energy (ORTN), National Research and Innovation Agency (BRIN), South Tangerang, Indonesia; 4 Department of Mechanical Engineering, Gunadarma University, Depok, West Java, Indonesia; 5 Center for Clinical Toxicology and Environmental Health, Faculty of Medicine, Diponegoro University, Semarang, Indonesia; 6 Environmental Health Department, Faculty of Public Health, Hasanuddin University, Makassar, Indonesia; 7 Institute of Radiation Emergency Medicine, Hirosaki University, Aomori, Japan

**Keywords:** annual effective dose, NORM, occupational radiation exposure, public health, radiation protection

## Abstract

The tin industry in Indonesia is a major global producer, and mining and smelting processes can mobilize and concentrate Naturally Occurring Radioactive Materials (NORM), particularly radionuclides from the ^238^U and ^232^Th decay series, potentially leading to occupational radiation exposure. We conducted a comprehensive assessment of annual effective doses to workers by integrating external and internal exposure pathways at two tin smelters using raw materials with different tin contents. External exposure was evaluated using ambient dose equivalent rate measurements, while internal exposure was assessed through simultaneous measurements of radon (^222^Rn), thoron (^220^Rn), and thoron progeny concentrations with passive discriminative detectors. Activity concentrations of ^226^Ra, ^232^Th, and ^40^K in raw materials and by-products were determined by HPGe gamma spectrometry to identify dominant contributors to gamma radiation fields. The total annual effective dose for most workers was <2 mSv, but reached 20.72 mSv for slag handling at one site, exceeding the recommended IAEA occupational limit. Radionuclides from the ^232^Th decay chain were the dominant contributors to external gamma exposure, particularly in slag, with activity concentrations increasing from raw materials to slag by approximately 28-fold for ^226^Ra and 79-fold for ^232^Th at Smelter A. Internal exposure was mainly influenced by thoron progeny in smelting areas, whereas radon contributed more in office and laboratory environments. These results identify slag storage as a critical radiological hotspot and highlight the need to evaluate both exposure pathways to support ALARA-based protection strategies and regulatory frameworks for NORM management in industrial settings.

## 1 Introduction

Natural resource developments, including mining, refining, and waste management, are the basis of a substantial portion of the global economy and provide the raw materials for much of the infrastructure and tools used in daily life (1, 2). One industrial activity involving naturally occurring radioactive sources is tin mining and smelting on Bangka Island, Indonesia. Tin ores contain multiple chemical constituents, including rare earth elements (REEs) and may contain naturally occurring radioactive material (NORM), such as uranium, thorium, and their radioactive progeny, as by-products (3, 4). This major industry employs a large workforce in an environment where NORM can be mobilized and concentrated within process streams (5).

The tin industry in Indonesia has a history spanning more than 200 years and is currently the second-largest tin producer in the world, with an annual production of approximately 50,000 tons (4, 6, 7). As with many metallic ores, tin deposits commonly contain multiple chemical elements. Consequently, mining and smelting activities can result in the release and concentration of radionuclides and heavy metals, leading to radiation exposure of workers, the public, and the surrounding environment.

The tin smelting industry on Bangka Island, Indonesia, represents a significant NORM-related activity, processing ores that contain primordial radionuclides from the ^238^U and 232th decay series, as well as ^40^K. Previous studies in Bangka Island have shown that ambient radiation levels are approximately twice the global average (2.4 mSv y^−1^), with the maximum background dose rates reaching 596 nSv h^−1^ in Muntok City, the location of the Pemali tin deposit (6). Other studies have reported potential environmental risks associated with the storage of tin-smelting by-product (6, 8–10). In addition to environmental concerns, tin smelting poses potential occupational radiation exposure. Workers may be exposed to external gamma radiation from radionuclides in the ores, intermediate materials, and slag. Internal exposure may occur through inhalation of radon (^222^Rn) and thoron (^220^Rn) released into the workplace air, as well as through airborne particulates caused during high-temperature processing and from stored materials. Given the large workforce employed in this industry, where NORM can be released and concentrated during processing, both external and internal radiation exposures, particularly from radon and thoron progeny, are of relevance for workers directly involved in smelting operations.

Issues related with NORM-related industries are particularly pronounced in tin-producing regions such as Bangka Island, where economic dependence on tin mining coexists with high levels of environmental radiation exposure and complex waste management challenges. To date, no published studies have comprehensively evaluated occupational radiological exposure among workers in the tin-smelting industry in Indonesia. This study addresses this gap by providing the first integrated assessment of annual radiation doses received by tin-smelting workers, including a range of occupational roles from administrative staff to personnel directly involved in production processes. The assessment integrates external gamma doses with internal exposure from thoron progeny in high-temperature smelting environments and evaluates the distribution of radiation doses across multiple exposure pathways for distinct worker categories in the NORM industry. Measurements were conducted across key operational areas, including raw-material storage, smelting, and post-processing zones, as well as slag and waste-handling facilities, using two representative tin sources. By identifying and characterizing radiation-exposure points associated with NORM enrichment during high-temperature processing, this study clarifies potential impacts on both workers and the surrounding environment.

This study provides important baseline data to support improved occupational radiation protection, application of the As Low As Reasonably Achievable (ALARA) principle, and the development of national policies and regulatory frameworks for NORM and industrial-waste management. In this context, the study is aligned with the United Nations Sustainable Development Goals (SDGs) to advance sustainable development and benefit humanity. This research focuses on good health and well-being (SDG 3), clean water and air (SDG 6), industrial innovation and infrastructure (SDG 9), responsible consumption and production (SDG 12), and life on land (SDGs 14 and 15).

## 2 Materials and methods

### 2.1 Study area

Bangka Belitung Province contributes approximately 90–95% of Indonesia’s tin production, equivalent to 65,000–70,000 tons per year (11, 12). Approximately 76% of workers in the tin mining industry between 2004 and 2013 were local residents (13). This study categorized workers into several occupational types to determine the spread of radionuclides caused by this industry and to clarify its impact on the environment and surrounding communities.

**Figure 1:**
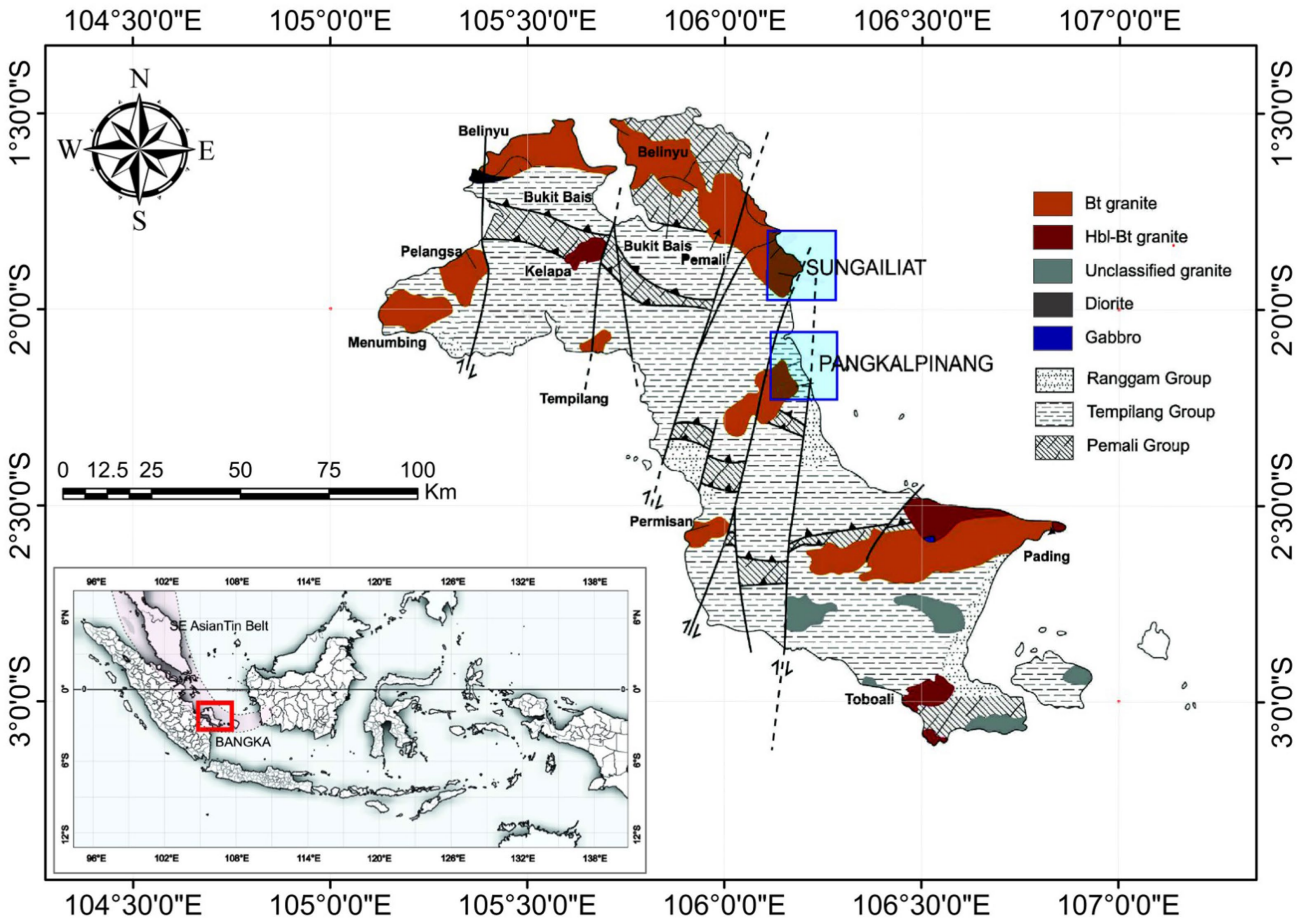
Geological map of Bangka Island, Indonesia and the study locations in Sungailiat (Smelter A) and Pangkalpinang (Smelter B).

The study was conducted at two tin smelter industries located in two major cities on the Bangka island, Sungailiat and Pangkalpinang. Sungailiat city has Smelter A located at S 1.8680388, and E 106.1069149, while Pangkalpinang city has Smelter B located at S2.1410465, and E106.1133337 (Figure 1). Smelter A is located in a rural area with a population of 95,427 people in 2023. Meanwhile, Smelter B is located near a city with a population of 236,267 people (14, 15). Maps were created by using the software ArcGIS version 10.8, derived from Geological Agency.

These two smelters represent the tin-processing industry in the region, which handles significant volumes of tin ore and produces various by-products. Each smelter uses a different type of raw material: in Smelter A, tin ore is used, while Smelter B uses byproduct slag, which still contains 20% tin. Measurement locations at each smelter were carefully selected to represent the potential areas for highest radiation levels. These areas include raw material storage, processing areas, slag storage areas, product storage areas, and temporary waste disposal sites.

**Figure 2:**
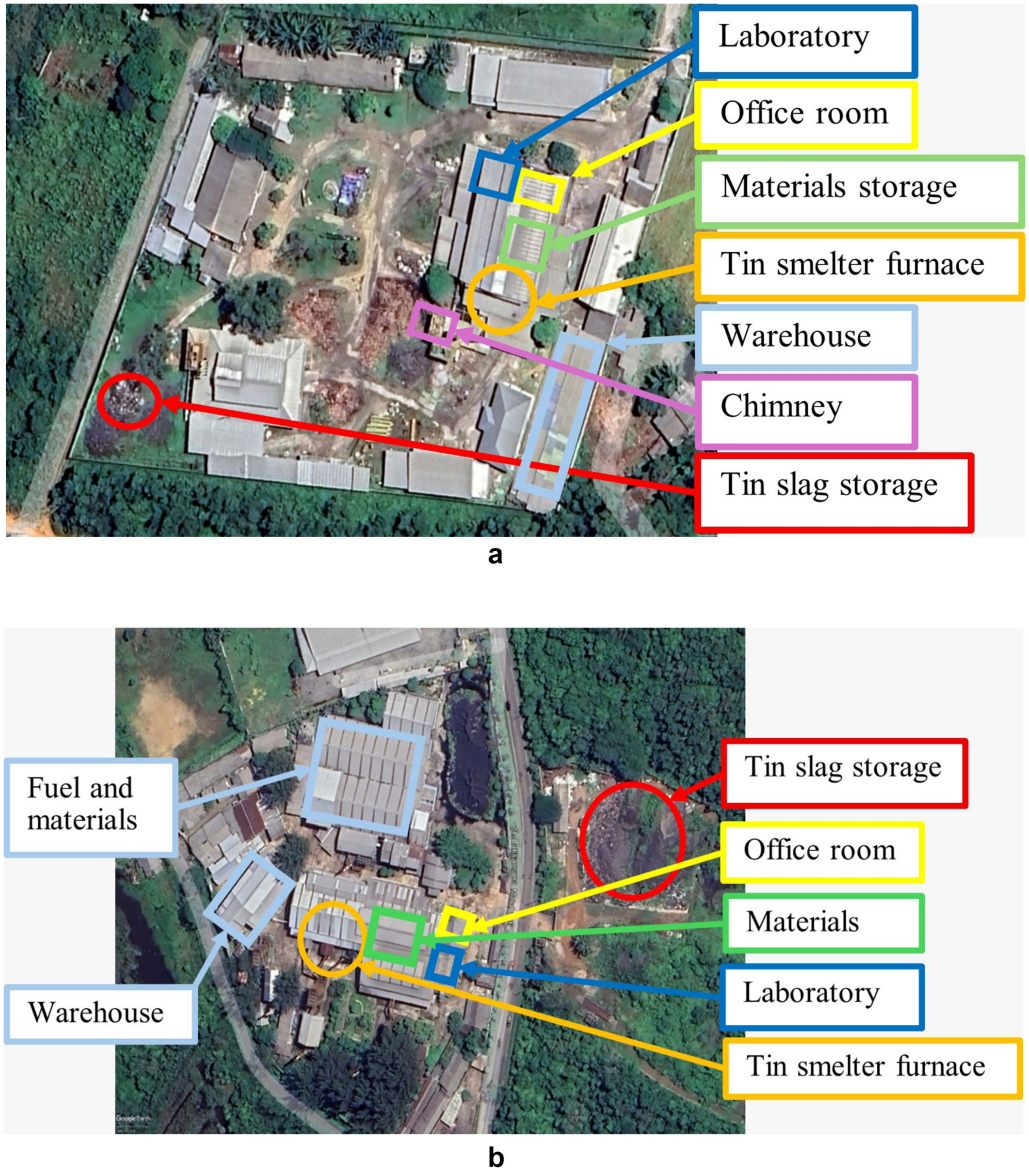
Location of facilities at **(a)** Smelter A and **(b)** Smelter B.

Smelter A consists of an administration building and a laboratory on the second floor, with the smelting area in front of the building (Figure 2a). Meanwhile, Smelter B consists of an administration building and a laboratory on the ground floor near the main entrance, and the smelting location is far behind the building (Figure 2b).

In the tin smelting process, the raw material is melted at high temperatures several times in a furnace to maximize tin recovery. The tin smelting process involves adding other materials, such as anthracite and flux, in specific ratios. Anthracite acts as a high carbon reducing agent to convert tin oxide into molten tin, while limestone acts as a flux to combine with impurities to form a slag that floats on top of the tin. Fuel, air, and oxygen injection are also regulated to maintain controlled conditions. This smelting process takes place at 1150 °C and produces crude tin with a content of > 80% in the bottom layer (16). The next stage is the reduction process, where the slag from the previous smelting process is then reduced again with anthracite and a high number of additional reductants. Remelting is then carried out at a slightly higher temperature of 1,150–1,250 °C, producing a Sn/Fe alloy and a slag with a tin concentration of around 3% (17, 18).

### 2.2 Comprehensive radiation measurement

a External exposure measurement

**Figure 3:**
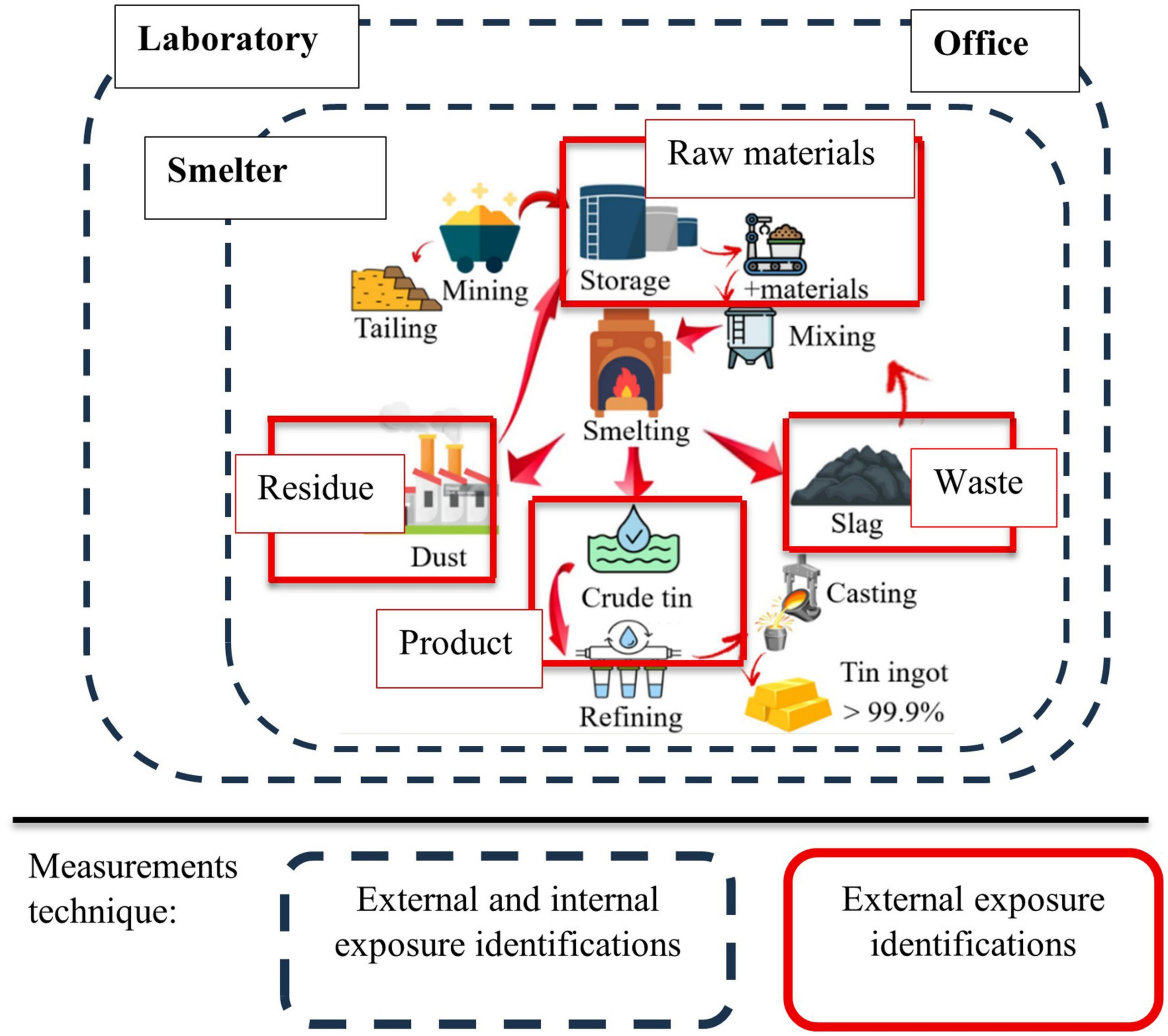
An external and internal exposure measurement scheme to identify the exposure received by workers in the tin smelting industry.

In this study, external radiation exposure and its contribution from different operational activities within the NORM industry were systematically evaluated. Measurements were conducted across three representative occupational categories: office, laboratory, and field work, which are most likely to be influenced by nearby mineral-processing operations. The external exposure was conducted ambient dose equivalent rate measurement using a CsI(Tl) scintillator detector survey meter with dimensions approximately 60(W)×27(D)×120(H)mm (Fuji Electric, Japan). This survey meter detects X-ray gamma radiation with a measurement energy range from 40 keV to 6 MeV. Measurements were taken at a height of 1 meter above ground level around the tin smelter area and in the smelter area at each stage of the industrial process at both smelters (Figure 3).

b Internal exposure measurement

In this study, radon and thoron concentrations were measured simultaneously using a passive detector, namely the RADUET passive radon-thoron discrimination monitor (Radosys, Hungary) (19–21). The lower detection limits (DLs) of the RADUETs were determined to be 3 Bq m^−3^ for radon and 4 Bq m^−3^ for thoron (22). The RADUET was calibrated at Institute of Radiation Emergency Medicine (IREM), Hirosaki University (23, 24). Additionally, direct measurement of thoron progeny concentration is needed to accurately estimate the radiation dose caused by their radionuclides using a deposition rate monitor (passive monitors). To obtain tracks of alpha particles from thoron progeny, especially ^212^Po, it is necessary to construct an appropriate method. The method was the same method as previously reported thoron progeny measurement in public dwellings in Bangka (6).

The total 24 passive detectors and monitors were placed inside the smelter to measure outdoor ^222^Rn and ^220^Rn progeny after obtaining prior permits. A total of 14 passive detectors and monitors were installed in Smelter A, and 11 in Smelter B. In Smelter A, the installation was carried out in the smelter area (10 detectors), the office room (2 detectors), and the laboratory room (2 detectors). While in Smelter B, the installation was carried out in the smelter area (7 detectors), the office room (2 detectors), and the laboratory (2 detectors). The equipment was installed by hanging it at a height of 1 to 2 m above the ground and positioned in the middle of the room at least 50 cm from the wall surface according to the location of the workers. Meanwhile, installation in the smelting place is done by hanging it on the top of a pole or pillar. The monitor was installed two times in a year: September 2023 to May 2024 and May 2024 to November 2024. This is to obtain representative data in calculating the average annual dose, which is more stable and less influenced by short-term fluctuations. In both smelters, the storage area for the materials to be used is kept in an open space together near the smelter. The building studied was constructed of sand bricks held together with a mixture of cement, water, and sand, then coated with wall paint, and the floor was covered with cement tiles. Meanwhile, the foundations of the smelting plants and floor are typically a mixture of gravel and cement, with steel walls used on some sides. Inside the smelter, there are several pillars to support the roof to keep it sturdy.

After sampling for the passive detector and monitor is completed, the CR-39 chip is analyzed by etching to determine the number of alpha tracks. The etching process is carried out according to each manufacturer’s specifications. In the Radosys product, Hungary, etching is carried out using a 6.25 M NaOH solution at 90 °C for 6 h, while in the Nagase Landauer, Ltd. Japan product etching is carried out using a 6 M NaOH solution at 60 °C for 24 h. The etching process increases the size of the tracks caused by alpha particles in CR-39, so they can be read and quantified under a microscope. Calculation of radon ( ${\bar{C}}_{\mathrm{Rn}}$ ) and thoron ( ${\bar{C}}_{\mathrm{Tn}}$ ) concentrations from the track in RADUET using Equations 1, 2 (6, 21, 22, 25, 26):


$$\begin{array}{l} {\bar{C}}_{\mathrm{Rn}}=(d_{L}-\bar{b})\times \frac{f_{\mathrm{Tn}2}}{t(f_{\mathrm{Rn}1}\times f_{\mathrm{Tn}2}-f_{\mathrm{Rn}2}\times f_{\mathrm{Tn}1})}\\ -(d_{H}-\bar{b})\times \frac{f_{\mathrm{Tn}1}}{t(f_{\mathrm{Rn}1}\times f_{\mathrm{Tn}2}-f_{\mathrm{Rn}2}\times f_{\mathrm{Tn}1})} \end{array}$$ (1)

$$\begin{array}{l} {\bar{C}}_{\mathrm{Tn}}=(d_{H}-\bar{b})\times \frac{f_{\mathrm{Rn}}}{t(f_{\mathrm{Rn}1}\times f_{\mathrm{Tn}2}-f_{\mathrm{Rn}2}\times f_{\mathrm{Tn}1})}\\ -(d_{H}-\bar{b})\times \frac{f_{\mathrm{Rn}}}{t(f_{\mathrm{Rn}1}\times f_{\mathrm{Tn}}-f_{\mathrm{Rn}}\times f_{\mathrm{Tn}})} \end{array}$$ (2)

Where $d_{L}$ and $d_{H}$ are the alpha track densities (track cm^−2^) in the CR-39 from the low and high air exchange rate chambers, respectively. $f_{\mathrm{Rn}}$ and $f_{\mathrm{Tn}1}$ are the ^222^Rn and ^220^Rn calibration coefficients (tracks m^−2^ kBq^−1^ m^3^ h^−1^) in the low air exchange rate chamber, respectively. $f_{\mathrm{Rn}}$ and $f_{\mathrm{Tn}2}$ are the ^222^Rn and ^220^Rn calibration coefficients in the high air exchange rate chamber (tracks m^−2^ kBq^−1^ m^3^ h^−1^), respectively. t is the exposure time in hours, and $\bar{b}$ is the background track density of the CR-39 detector. The concentration calculation for Equivalent Equilibrium Thoron Concentration ( EETC ) is based on the track counted using the Equations 3, 4:


$$N_{\mathrm{TnP}}=\text{EETC}\times CF_{\mathrm{TnP}}\times T+N_{B2}$$ (3)

$$\mathrm{EET}C=\frac{N_{\mathrm{TnP}}-N_{B2}}{CF_{\mathrm{TnP}}\times T}$$ (4)

Where $N_{\mathrm{TnP}}$ and $N_{B2}\;$ is the track density in the thoron progeny deposition detector that was installed and from the monitor that was isolated as background, EETC is the equilibrium equivalent thoron concentration, and $CF_{\mathrm{TnP}}$ is the conversion factor for thoron progeny which was (tracks m^−2^ kBq^−1^ m^3^ h^−1^). T is the duration of the monitor installation (6, 21). Calculation of uncertainty in radon and thoron concentrations in RADUET and EETC , using the equations previously reported by Pradana et al. (6). Furthermore, for quality control, the ^222^Rn and ^220^Rn monitor has been calibrated in the Institute of Radiation Emergency Medicine, Hirosaki University (23).

Inhalation of short-lived solid decay products will deliver most of the radiation dose to the human lung. So, the estimated dose using the EETC is based on direct measurements of thoron progeny. The equilibrium factor between radon, thoron, and their progeny determines the radioactive equilibrium level. The thoron equilibrium factor ( $F_{\mathrm{Tn}}$ ) was derived from field data in this study using the Equation 5 (27).


$$F_{\mathrm{Tn}}=\frac{\text{EETC}}{C_{\mathrm{Tn}}}$$ (5)

Where $C_{\mathrm{Tn}}$ is the thoron gas concentration in Bq m^−3^.

c Radionuclide measurements

The activity concentration of samples was measured using an HPGe detector (GEM 60–5, ORTEC, USA). This detector has a resolution of 1.81 keV at 1.33 MeV and a relative efficiency of 60%. The detector also has ultra-low background shielding using an aged lead-containing resist (ISuS, Sweden) used to measure ^226^Ra, 232th, 228th, ^40^K and enclosed in a compact 10 cm thick lead shield (28, 29). The counting system must be calibrated using a standard source in the same geometry as the sample. To prepare such a standard, a known amount of standard reference material (SRM) was filled into vials identical to the sample vials (30, 31).

Sample calculations were obtained by analyzing spectra from Multi Channel Analyzer (MCA) on a PC using Gamma Vision software (ORTEC, United States). The sample calculation time is about 80,000 counting times to obtain adequate uncertainty statistics. An estimate of the potassium concentration was obtained by detecting 1,460 keV gamma rays emitted by ^40^K. An Uranium estimation was performed through the detection of 609 keV gamma rays from ^214^Bi and 351 keV gamma rays from ^214^Pb, a product of the ^238^U decay series. Similarly, thorium estimation was performed through the detection of 581 keV gamma rays from ^208^Tl, 238 keV from ^212^Pb, and 911 keV from ^228^Ac, a product of the 232th decay series (4, 28, 32). The response of a gamma-ray instrument to radiation from K, U, or Th progeny source depends on the source concentration, the detector volume and efficiency, and the instrument’s energy threshold (33). We used Equations 6–8 to calculate the activity concentrations ( A ), uncertainty ( Uc ), and detection limit ( $L_{D}$ ) from these measurements, respectively (4, 28, 32, 34, 35).


$$A\;(\mathrm{Bq}\;kg^{-1})=\frac{\mathrm{CPS}}{E\times Y\times W\times f_{c}}\pm k\times U_{c}$$ (6)

$$\mathrm{Uc}=\sqrt{\sum U_{i}^{2}}$$ (7)

$$L_{D}=L_{C}+K\sigma_{C}$$ (8)

Where CPS is the number of counts per second, E is the efficiency, Y is the energy yield of the radionuclide, W is the sample weight (kg), and $f_{c}$ is the correction factor (including summing in, summing out, decay factor, recovery factor, attenuation factor, branching ratio, and growth factor). k is the coverage factor in k = 1.96 for a 95% confidence interval, $U_{c}$ is the combined uncertainty of the measurements, and $U_{i}$ is the absolute uncertainty of each relevant standard uncertainty. $L_{D}$ is the detection limit, $L_{C}$ is the critical level below which no signal can be detected, $\sigma_{C}$ is the standard deviation, and K is the error probability. The detection limits for ^40^K, ^226^Ra, and ^232^Th are 0.21 Bq kg^−1^, 0.08 Bq kg^−1^, and 0.08 Bq kg^−1^, respectively.

The absorbed dose rate in air can be evaluated from the activity concentration of radionuclides in a homogeneously distributed sample. The activity concentrations of radionuclides from the U and Th decay series will be the same. From this, the absorbed dose rate in air ( $D_{\mathrm{air}}$ ) can be estimated based on calculations of the conversion factors for each radionuclide by using Equation 9 (4, 26).


$$D_{\mathrm{air}}=(0.462\times A_{U})+(0.604\times A_{\mathrm{Th}})+(0.0417\times A_{K})$$ (9)

Where $D_{\mathrm{air}}$ is the absorbed dose rate in the air (nGy h^−1^), $A_{U}$ , $A_{\mathrm{Th}}$ , and $A_{K}$ are the activity concentrations of ^226^Ra, ^228^Ra and ^40^K, respectively, in Bq kg^−1^. The coefficients of 0.462, 0.604 and 0.0417 are conversion factors (absorbed dose rate in air per unit activity per unit of sample mass, in units of nGy^−1^ per Bq kg^−1^) evaluated for ^238^U-series, ^232^Th series and ^40^K, respectively (4, 26).

d Annual effective dose calculation

The annual effective dose calculation is performed to evaluate the risk of radiation exposure in the workplace. These effective dose estimates are used to determine the highest dose exposure in the workplace. This calculation is performed by accumulating the effective doses from internal exposure and external exposure. Estimation of the annual effective dose (mSv) from indoor radon ( $D_{\mathrm{Rn}}$ ) and thoron ( $D_{\mathrm{Tn}}$ ) in workplace using Equations 10, 11, respectively:


$$D_{\mathrm{Rn}}=C_{\mathrm{Rn}}\times EF_{\mathrm{Rn}}\times DF_{\mathrm{RnP}}\times T$$ (10)

$$D_{\mathrm{Tn}}=\text{EETC}\times DF_{\mathrm{TnP}}\times T$$ (11)

Where $C_{\mathrm{Rn}}$ and EETC are the activity concentrations of ^222^Rn and ^220^Rn progeny in Bq m^−3^ from passive detector measurements. $EF_{\mathrm{Rn}}$ is the radon equilibrium factor in indoor environments with a value of 0.4 according to the United Nations Scientific Committee on the Effects of Atomic Radiation (UNSCEAR) and International Commission on Radiological Protection (ICRP) (36, 37). *T* is the working time, with 2080 h year^−1^. $DF_{\mathrm{RnP}}$ and $DF_{\mathrm{TnP}}$ are inhalation dose conversion factors with values 1.7 × 10^−5^ mSv (Bq h m^3^) and 1.07 × 10^−4^ mSv (Bq h m^−3^)^−1^ for ^222^Rn and ^220^Rn, respectively (37).

Meanwhile, the estimation of the annual effective dose (mSv) from external gamma radiation ( H) is based on the measurement of the ambient dose equivalent rate. Calculation of H using Equation 12:


H=D×T×Cf (12)

Where D is the external gamma dose rate (nSv h^−1^), T is the working time, with 2080 h year^−1^. This is calculated by multiplying the 52 weeks in a year by 40 working hours per week based on Labour laws No 13 (38). Cf is conversion factor 0.6 (6). The total annual effective dose ( $\mathrm{AE}D_{\text{Total}})\;$ from internal and external exposure is calculated per the Equation 13:


$$\mathrm{AE}D_{\text{Total}}=D_{\mathrm{Rn}}+D_{\mathrm{Tn}}+H_{\text{external}}$$ (13)

## 3 Result and discussion

### 3.1 Characterization of NORM in materials

Gamma radiation exposure is a significant pathway for workers in mineral processing environments, especially tin smelting. This gamma radiation exposure comes from ^40^K and the ^238^U and ^232^Th, decay chains, where these radionuclides emit alpha, beta, and gamma rays. Workers can receive external and internal exposure; the closer to the source, the greater the gamma exposure received. For this reason, measurements were carried out on raw materials and by-products to determine the gamma-emitting source’s activity concentration. The dominant gamma emitters are short-lived decay daughters in the ^238^U decay chain; ^214^Pb and ^214^Bi from ^226^Ra, while in the ^232^Th decay chain are ^228^Ac, ^212^Pb, and ^208^Tl.

The activity concentration values for each dry material are shown in Table 1. The results of the calculation of the highest absorbed dose rate from the activity concentration are 16,943 nGy h^−1^ and 11,072 nGy h^−1^ in both slags. In addition, it is also found in the raw material at smelter B at 8,672 nGy h^−1^, which includes slag material (Slag 1) originating from other industries. The absorbed dose rate is within the range reported for other Tin slag measurements on Bangka Island, around 8,631–21,104 nGy h^−1^. This shows that the average absorbed dose rate in tin slag falls within that range, depending on the geological conditions used for raw material extraction.

**Table 1:** Distribution of absorbed dose rate from the calculation of activity concentration.

Area	Activity concentration (Bq kg^−1^ dry)									Absorbed dose rate
	^226^Ra			^232^Th			^40^K			(nGy h^−1^)
Smelter A										
Raw material	334	±	8	265	±	7	17	±	3	315
Anthracite	4.8	±	0.4	11	±	1	41	±	2	10
Flux	10	±	1	≤ LLD			1.7	±	0.2	5
Dust	95	±	3	170	±	5	120	±	5	152
Crude tin	1.4	±	0.1	5	±	1	≤ LLD			3.44
Slag	9,187	±	205	20,930	±	454	1,382	±	42	16,943
Smelter B										
Raw material	4,949	±	110	10,515	±	227	817	±	24	8,672
Anthracite	22	±	2	41	±	3	59	±	5	37
Flux	65	±	2	112	±	4	16	±	1	98
Dust	379	±	9	846	±	19	163	±	7	693
Crude tin	150	±	5	348	±	9	25	±	2	280
Slag	5,784	±	131	13,845	±	302	887	±	32	11,072

Based on the activity concentration reported in Table 1, substantial enrichment was observed from raw materials to the final slag-storage stage at Smelter A, with activity concentrations increasing by approximately 28-fold for ^226^Ra and 79-fold for ^232^Th. This enrichment reflects differences in feedstock characteristics: at Smelter A, the raw material consists of tin ore concentrate, whereas at Smelter B the feedstock comprises slag from other smelters that still contains approximately 20% residual tin.

**Figure 4:**
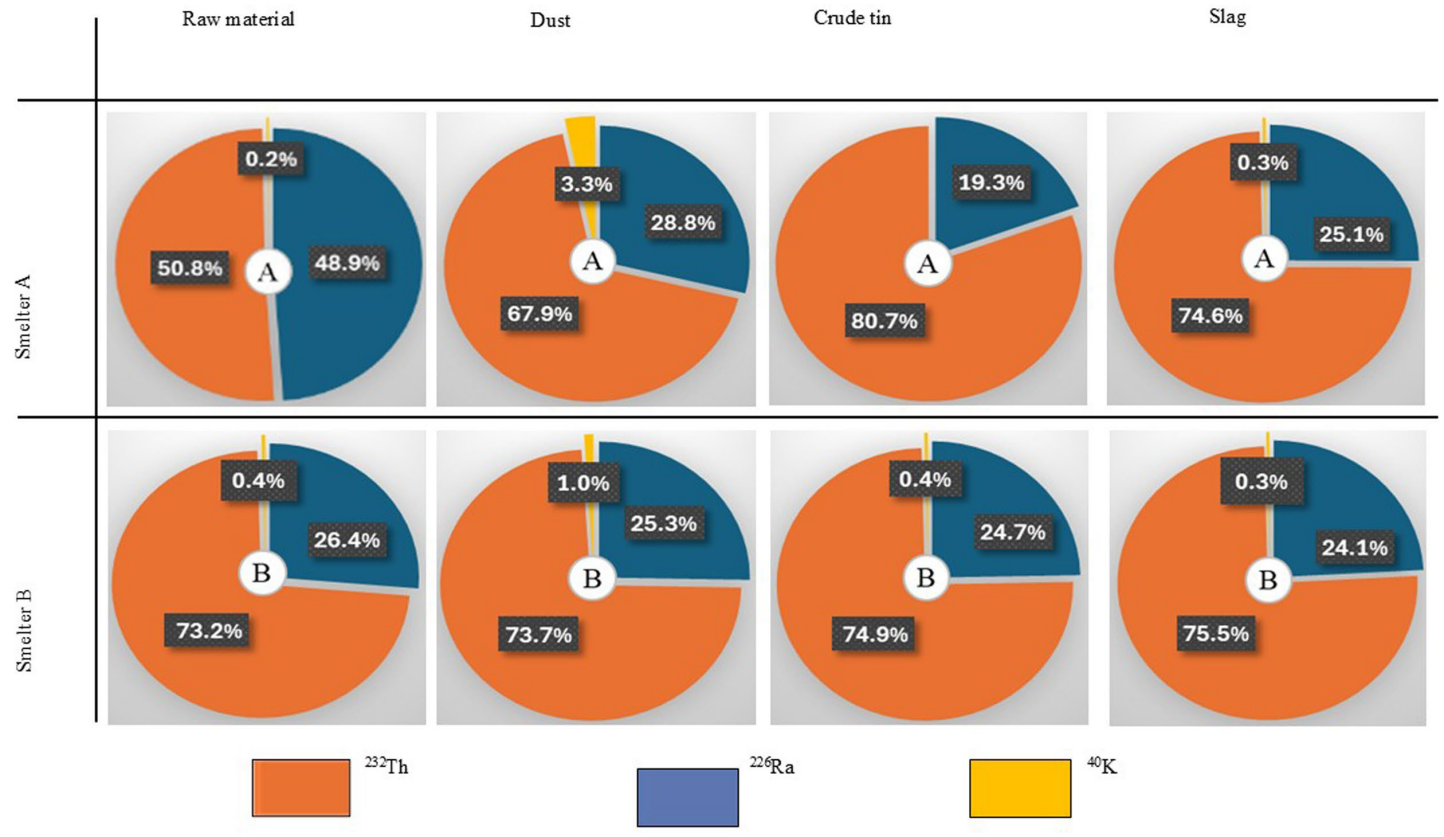
Percent contribution of radionuclides to dose equivalent rate at smelters.

The relative contributions of 232th, ^226^Ra, and ^40^K to the absorbed dose rate are shown in Figure 4. In slag samples, ^232^Th was the dominant contributor, accounting for74.6 and 75.5% of the absorbed dose rate at Smelter A and B, respectively.

### 3.2 External exposure field

The tin smelting process has the potential expose workers to ionizing radiation from gamma-emitting radionuclides present in the chimney, intermediate products, and slag. Results showed that radiation exposure levels in smelting areas were consistently higher than those measured in offices and laboratories, with ambient dose equivalent rates ranging from 120 to 570 nSv h^−1^. In Smelter A, the mean ambient dose equivalent rate was approximately twice that observed at Smelter B of 338 nSv h^−1^. This situation is caused by two smelters handling different material concentrations, where smelter A uses 60–75% tin raw material, while smelter B uses 20% tin raw material from the previous tin smelter. Smelter A extracts more tin metal than smelter B, so the NORM enrichment in the Smelter A area is higher than in the Smelter B area. Meanwhile, radiation exposure in offices and laboratories varies depending on their location relative to the smelter. The ambient dose equivalent rate measurements for both smelters during operation are shown in Table 2.

**Table 2:** The ambient dose equivalent rate measurements during operation.

Area	N	Ambient dose equivalent rate (nSv h^−1^)			
		Average (Value ±SD)			Range
Smelter A					
Smelter	10	338	±	141	120–570
Office room	2	165	±	5	160–170
Laboratory	2	175	±	5	170–180
Storage					
- Raw material	2	206	±	30	176–236
- Anthracite	2	166	±	15	151–181
- Flux	2	124	±	10	114–134
- Dust	2	797	±	30	767–827
- Crude tin	2	178	±	20	158–198
- Slag	2	3,150	±	90	3,060-3,240
Smelter B					
Smelter	7	160	±	31	120–210
Office room	2	75	±	25	50–100
Laboratory	2	175	±	45	130–220
Storage					
- Raw material	2	218	±	40	178–258
- Anthracite	2	124	±	20	104–144
- Flux	2	94	±	25	69–119
- Dust	2	227	±	35	192–262
- Crude tin	2	73	±	35	40–110
- Slag	2	10,070	±	110	9,960–10,180

This range of ambient dose equivalent rate measurements in the smelter area by using a survey meter is slightly higher than the survey measurements around Bangka Island by using car-borne survey, which ranged from 29 to 595 nSv h^−1^, and the survey in 2015 with spot measurement ranging from 44 to 512 nGy h^−1^ (4, 39). The average values of 338 nSv h^−1^ and 160 nSv h^−1^ for both smelters area in the present study are higher than the average values of 103 nSv h^−1^ and 133 nGy h^−1^ in the surveys conducted in 2023 and 2015, respectively. Also, laboratory and office spaces have higher average values than public exposure. Regarding the ambient dose equivalent rate around the tin smelter, it can be clarified that the smelter area has the highest gamma radiation exposure due to the NORM enrichment process, rather than the public area.

The ambient dose equivalent dose rate distribution source was also performed on materials before the smelting process and on post-smelting products. Exposure in the raw material area was around 69–258 nSv h^−1^; meanwhile, exposure after smelting, in the product of crude tin and tin dust, was 40–827 nSv h^−1^. However, in the slag storage area, it was identified as much higher than other workplace areas, with a range of 3,060–10,180 nSv h^−1^. These are due to its physical and chemical properties, which make it more stable as an unreduced oxide. The enrichment of tin slag metal oxides indicated an increase in uranium and thorium contents (40). This situation is due to the slag from Smelter B being processed multiple times. Therefore, the NORM enrichment in this slag is greater than that at Smelter A. This results in NORM enrichment being higher in Smelter B, which aligns with the highest tin metal recovery. The average ambient dose equivalent rate values in slag storage at both smelters indicate an increase in NORM and require special attention for radiation protection.

The ambient dose equivalent rate for raw materials before processing is slightly higher than that in the public, because the raw materials are selected and concentrated through physical and chemical separation processes. In addition, raw material storage is conducted in a semi-open area at the same location, leading to ambient equivalent dose rate measurements that influence one another. Meanwhile, in the dust storage at Smelter A, the value is 3.3 times greater than at Smelter B.

Collectively, these data demonstrate spatial variability in radiation levels, providing information about the potential risks of external radiation exposure. Variations in ambient dose equivalent rates reflect the combined influence of industrial processing activities, radionuclide composition and enrichment, material handling, and local environmental conditions within the workplace, resulting in heterogeneous exposure patterns. The spatial distribution of the above data reveals localized radiation hotspots, particularly in areas associated with mineral processing and waste storage, indicating a planned exposure situation.

According to the International Atomic Energy Agency (IAEA), planned exposure situations refer to occupational exposures where industrial activities involve radiation exposure involving NORM. Therefore, exposure control is guided by the principles of dose limitation, optimization (ALARA), and justification, thereby minimizing worker health risks through strict regulation. The effective dose limit for planned occupational exposure is 20 mSv y^−1^, averaged over five consecutive years (maximum cumulative 100 mSv), with a maximum limit of 50 mSv y^−1^ if necessary (41). This limit is designed to prevent tissue reactions in human tissue while minimizing stochastic risks through management, and is supported by long-term dose-optimization strategies, such as ALARA, for sustainable radiation protection in industrial environments associated with NORM.

The annual and cumulative average limits are designed based on scientific research on the relationship between the tissue reactions and stochastic effects of exposures approaching or exceeding 100 mSv (41). Scientific research on low doses below 100 mSv includes biochemical reactions, oxidative modifications of amino acid residues in serum albumin, and effects on protein structure and function, although not directly correlated with acute clinical effects (42–45). The health risks of low chronic exposure (≤100 mSv) are uncertain, so for planned exposure, caution is needed about the need for practical regulatory controls. By maintaining cumulative occupational doses below 100 mSv for 5 years and imposing annual limits, the regulatory framework ensures that workers remain within the safe exposure range.

Supplementary Table S1 lists the radionuclide concentration in soil in public areas and around tin mining sites, indicating environmental radionuclide exposure (6, 36, 46–48). From these references, it can be concluded that the highest radionuclide content is found in smelting sites, both in pre- and post-smelting materials.

### 3.3 Internal exposure concentration

Radon is the second leading cause of lung cancer after smoking, as reported by World Health Organization (WHO) and UNSCEAR (49, 50). This is based on epidemiological studies in several countries, where the risk increases by 16% for every 100 Bq m^−3^ of Radon (6). The limit of Indoor radon reference of 300 Bq m^−3^ in the Workplace from regulations issued by the Indonesian government (51). The ICRP also recommends a reference value of 300 Bq m^−3^ for radon for protection purposes in homes and workplaces (52).

To understand the health risks in high-temperature industrial environments where U, Th, and K-rich materials are processed, it is also important to recognize the local, non-uniform exposure to ^220^Rn disequilibrium and its decay products in the air. This will result in particles adhering to aerosols that will be inhaled and deposited in the respiratory tract until dynamic equilibrium is reached in the lungs. In the identified tin smelting area, the average indoor thoron equilibrium factor was higher than in the smelting area, exceeding 0.040, the value typically used by UNSCEAR (36), except in smelting area A. Variations in the thoron equilibrium factor may be due to the inhomogeneous spatial distribution of thoron and the homogeneous distribution of thoron decay products (53), influenced by environmental conditions such as distance from the source, temperature, ventilation, and aerosol concentration. The average annual of ^222^Rn and ^220^Rn progeny and thoron equilibrium factor in both smelting areas has been shown in the Table 3.

**Table 3:** ^222^Rn and ^220^Rn progeny and $F_{\mathrm{Tn}}$ equilibrium factor.

Area	N	Annual average (Bq m^−3^)						Thoron equilibrium factor	
		Radon			EETC			Range	Average
Smelter A									
Smelter	10	21	±	4	1.35	±	0.54	0.01–0.05	0.02
Office room	2	23	±	2	0.86	±	0.18	0.03–0.06	0.04
Laboratory	2	36	±	2	0.51	±	0.03	0.06–0.07	0.07
Smelter B									
Smelter	7	16	±	4	1.26	±	0.68	0.03–0.17	0.07
Office room	2	17	±	4	0.84	±	0.43	0.01–0.19	0.1
Laboratory	2	16	±	2	1.46	±	0.3	0.03–0.07	0.05

In this study Supplementary Table S2, measurements of ^222^Rn and ^220^Rn progeny were conducted twice a year; May to November represents the dry season, and November to June represents the rainy season. This was done to determine the influence of weather on the distribution of ^222^Rn and ^220^Rn gases, which can result in inhalation exposure (54). This study showed that the concentrations of ^222^Rn and ^220^Rn progeny did not differ significantly between the two seasons. The results of the paired t-test show the probabilities (Table 4). This is due to the tropical climate on Bangka Island, which results in very little temperature difference between the dry and rainy seasons (6).

**Table 4:** The probability of no difference between two seasons.

*p*-value	Smelter area		Indoor area (Lab & Office)	
	A	B	A	B
Radon	0.99	0.09	0.18	0.34
EETC	0.47	0.18	0.15	0.8

The annual average radon concentration at the smelter site with a large area and semi-open space is lower than indoor office and laboratory locations, at around 15.78–20.80 Bq m^−3^. However, inhalation exposure of thoron progeny is higher, at 1.26–1.35 Bq m^−3^ in the smelter area. Adequate ventilation in the smelting area allowed the ^222^Rn and ^220^Rn released into the air to be directly released to the outside environment. Meanwhile, the ^220^Rn progeny concentration in smelter A, which uses ~70% tin as raw material, was identified as higher than in other workplace areas. At high temperatures, thermal expansion and increased gas diffusion can occur in materials, allowing very short-lived ^220^Rn (~55 s) to escape into the air before decaying within the material. Consequently, its decay products (^212^Pb and ^212^Bi) rapidly attach to aerosols (27, 55, 56). They are easily inhaled by workers near the source, thus delivering significant doses to the respiratory tract. Furthermore, the installation of passive monitors and different building construction (57), resulting in high ^220^Rn progeny concentrations around the smelter area.

In contrast to indoor ^222^Rn concentrations such as offices and laboratories, which have slightly higher values due to the accumulation effect in closed spaces compared to semi-open smelting areas, where the use of air conditioning is more common in these rooms, so that the differences in ^222^Rn concentrations between rooms vary slightly, although not significantly, because the intensity of opening and closing the room causes radon concentrations to vary slightly. Meanwhile, for ^220^Rn progeny indoors, which is slightly lower in November–June, because most of the ^220^Rn decay will quickly attach to ambient aerosols immediately after decay, creating radioactive aerosols (55), so that rainfall tends to reduce ambient in the air due to low PM2.5 concentrations originating from outdoors (58, 59). The concentration of ^220^Rn progeny has a relatively uniform spatial distribution, mainly due to the longer half-life of ^220^Rn progeny (the half-life of ^212^Pb is 10.6 h), will accumulate longer indoors.

In comparison with Betare-Oya gold mining area (Cameroon) in 2016, the radon, thoron, and thoron derivative values were 88–282, 19–383, and 1–19 Bq m^−3^, respectively. Meanwhile, the thoron equilibrium factor in this study was within the range reported in Gold mining area, ranging from 0.01–0.55 (60). Meanwhile, compared with thoron equilibrium factor in the public areas of Franceville, Moanda, and Mounana, in the Gabon, it was in the range of 0.004–0.710; 0.005–0.750, and 0.006–0.794, respectively (61). Both locations are rich in NORM, especially the 232th radionuclide. Furthermore, the region has gold mining sites in Cameroon and uranium and manganese in Gabon, which enrich 232th during processing. Therefore, the behavior of radon and its progeny is comparable in both areas. However, compared to similar areas on Bangka Island and throughout Bangka Island, indoor radon concentrations are lower or nearly the same as those in public areas (Supplementary Table S3). Nevertheless, the thoron progeny in the smelting area is lower than the global average of 0.04 Bq m^−3^ reported by UNSCEAR.

### 3.4 Integrated dose assessment

a Annual Effective Dose from external exposure

The highest estimated annual effective dose from external radiation exposure was around Smelter A, which was 1.12 mSv (Table 5), taken from the highest ambient dose equivalent rate exposure in each area. The difference in the estimated annual effective dose between the two smelters can be attributed to several factors, including variations in the geological origin of the tin ore, differences in the efficiency of the smelting process in concentrating radionuclides, and differences in material storage practices.

**Table 5:** The distribution of annual effective dose from external exposure.

Area	*H* (mSv)	
	Smelter A	Smelter B
Smelter	1.12	0.42
Office	0.33	0.12
Lab	0.35	0.27
Storage		
- Raw material	0.37	0.37
- Anthracite	0.31	0.22
- Flux	0.24	0.15
- Dust	1.6	0.4
- Crude	0.33	0.14
- Slag	6.36	20.72

From two smelters observed, the highest estimated annual effective dose was at the slag storage of Smelter B, which exceeded the occupational exposure limit recommended by the IAEA of 20 mSv (62). The high estimated annual effective dose level in the slag storage area of Smelter B is 3.3 times higher than that of Smelter A. This is due to the amount of slag in Smelter B being greater than that of Smelter A, hence the slag storage in Smelter B is made separate from the workers. In addition, the high tin recovery process in raw tin leaves a tin content of 2–3%, in line with the increase of several important and hazardous materials in the slag phase, including primordial radioactive materials. Where in the smelting process there is a high reduction of FeO by the added reductant (anthracite), so that the dissolved Fe will precipitate as an Fe-Sn intermetallic phase and tends to float to the surface to be removed. The high dose rates in the slag storage areas at Smelter A and B make them critical areas requiring stringent radiation monitoring and control. These findings are consistent with previous research showing that industrial byproducts, particularly from the processing of tin containing minerals, can serve as reservoirs for high levels of naturally occurring radionuclides.

b Annual Effective Dose from internal exposure

The lung cancer risk from ^222^Rn and ^220^Rn inhalation stems primarily from their decay products, not from the particles themselves. Radon is more homogeneously distributed indoors than thoron (63), so inhalation dose assessments rely more heavily on radon equilibrium factors. In contrast, thoron gas is used only to correct for interference in radon measurements. Therefore, the effective dose from thoron inhalation is estimated using the EETC obtained by direct measurement of thoron’s decay products. By using the assumption of an occupancy factor of 0.2 and working hours of 2,080 h y^−1^, the highest annual effective dose from internal exposure is 0.89 mSv in the smelting area, while indoors it is around 0.56–0.66 mSv, as shown in Table 6. Several factors that influence this measurement are ventilation, humidity, and the influence of the building materials used.

c Calculation of Total Annual Effective Dose

**Table 6:** The distribution of annual effective dose from internal exposure.

Area	Radon contribution to inhalation dose (AM ± SD) (%)	Annual effective dose (mSv)
Smelter A		
Smelter	51 ± 11	0.89
Office	64 ± 5	0.56
Lab	82 ± 0.03	0.66
Smelter B		
Smelter	47 ± 11	0.89
Office	59 ± 8	0.57
Lab	41 ± 7	0.6

AM means Arithmetic Mean; SD means Standard Deviation.

Table 7 shows the total estimated annual effective dose from internal and external exposure at both smelters, with the most significant exposure occurring within the smelter area. Smelter A has a greater contribution from external exposure, reaching 57%, to the total annual effective dose, due to its high tin recovery, offset by its high enrichment in NORM and other metals. Therefore, the total estimated annual effective dose in the smelter, office, and laboratory areas is approximately 1.32–2.07, 0.78–0.91, and 1.04–1.06 mSv, respectively. These values are greater than the public exposure on Bangka Island and the world average of 0.7 and 0.48, respectively (4, 50).

**Table 7:** The distribution of contribution to annual effective dose.

Area	AED total (mSv)	Contribution (%)	
		External dose	Internal dose
Smelter A			
Smelter	2.01	57	43
Office	0.89	39	61
Lab	1.02	36	64
Storage			
- Raw material	0.37	-	NM
- Anthracite	0.31	-	NM
- Flux	0.24	-	NM
- Dust	1.60	-	NM
- Crude	0.33	-	NM
- Slag	6.36	-	NM
Smelter B			
Smelter	1.30	33	67
Office	0.70	27	73
Lab	1.88	43	57
Storage			
- Raw material	0.37	-	NM
- Anthracite	0.22	-	NM
- Flux	0.15	-	NM
- Dust	0.40	-	NM
- Crude	0.14	-	NM
- Slag	20.72	-	NM

*NM, Not measured.

As summary, for indoor work (laboratory and office workers), the risk of internal exposure is greater than that of external exposure. Several factors contribute to the high level of internal exposure, primarily radon in building materials and indoor ventilation. Meanwhile, the magnitude of external exposure in the smelting area depends on the field conditions that influence it, such as the placement of high-risk materials, such as raw materials, dust, and slag, around the smelting area. In addition, the estimated effective dose around the raw material storage is also shown in (Table 7), where the exposure in the raw material area is greatest in the slag storage area, where the slag storage area of smelter B has an effective dose exposure value greater than 20 mSv. In this study, internal exposure measurements were not conducted for each storage because material storage in both smelters was carried out in a single location simultaneously, making a more accurate identification of internal exposure unreliable. Therefore, the assessment of the contribution to the annual effective dose in the storage area cannot be compared between its external and internal exposure.

A comparison of the annual effective dose in Bangka Island and other areas is shown in Supplementary Table S4 (4, 46, 50, 60, 64–66). The annual effective dose in the smelter work area in this study ranged from 1.32 to 2.07 mSv y^−1^, which is higher than the annual effective dose in the public around the tin industry, the Indonesian average, and other mining areas except the gold mine in Cameroon. Furthermore, the indoor work area had an annual effective dose that was relatively higher than the public average in Bangka Island. The high estimated annual effective dose in the tin smelter area indicates that the area serves as a center for enrichment of minerals containing NORM, resulting in relatively high levels of environmental radiation exposure. The accumulation of radionuclides in slag, dust, and process residues has the potential to increase exposure to surrounding environmental components through airborne particle deposition and material transfer, both to workers at the smelter site and the environment. Therefore, strict distance regulation, the establishment of buffer zones, and continuous monitoring are needed to limit the spread of radionuclides and minimize radiological impacts on the surrounding environment.

As the result, the estimation of the total annual effective doses for all assessed worker roles remained below the 20 mSv y^−1^ occupational limit recommended by the IAEA (41), except for slag storage at smelter B. These findings underscore the complex dynamics of radiation dose rates from high radiation exposure identification and illustrate spatial variations; thus, this study provides insight into areas requiring more attention in the tin smelting area.

d Radiation Protection and Regulation

The large number of NORM residues resulting from past activities and Indonesia’s archipelagic geography present significant challenges for controlling NORM in industry. Strategic issues related to the NORM residues industry in Indonesia are expected to establish clear regulations for regulating industries involving NORM. Therefore, coordination between authorities is needed to build a national NORM management system in Indonesia. This regulation should be based on a phased approach, starting with Radiation Protection, Decontamination, the Circular Economy of NORM residues, Temporary storage of NORM residues/waste from the NORM industry, and final disposal of NORM waste (67).

Implementing worker protection strategies to ensure worker exposure remains as low as possible must utilize basic radiation protection principles, such as optimizing distance, time, and the use of shielding. Several practical recommendations for worker protection from radiation in industry-related NORMs that can be applied in the field:

1 Engineering controls by managing primary radiation sources from the storage of slag, residue, and NORM-rich raw materials in dedicated areas, implementing good ventilation to reduce concentrations of radon, thoron, and their derivatives, especially in high-temperature areas, and dust control to reduce contamination in the work area.
2 Administrative controls by limiting working hours, zoning work areas based on exposure levels, worker training, and implementing standard work procedures.
3 Use of Personal Protective Equipment (PPE): Special work clothing and respirators or particulate masks in areas with high inhalation risk.

Estimation of the total annual effective doses for workers in this workplace area is a preliminary study to identify and assess radiation doses. Subsequently, individual dosimeter monitoring will be conducted to estimate representative individual exposure. Planned monitoring approach, we plan to deploy individual Thermoluminescence Dosimeter (TLD) dosimeters for all workers, following established protocols such as Nugraha et al., who successfully quantified personalized exposure profiles in high background radiation areas of Mamuju, Indonesia (21). This approach will enable comprehensive dose reconstruction and enhanced occupational health surveillance.

These findings enable the implementation of optimized protection strategies—such as engineering controls for slag management, enhanced ventilation to mitigate airborne progeny, and administrative measures—to ensure exposures are maintained (ALARA). Considering the high effective dose rate of 20.72 mSv y^−1^ in the slag storage area, priority ALARA action recommendations include implementing standard work procedures, limiting time spent near the pile through job rotation, and installing permanent shielding. Furthermore, this evidence-based assessment offers valuable input for regulatory development on the management of NORM and NORM-containing wastes in industrial settings.

## 4 Conclusion

This study comprehensively examines the annual radiation doses received by various types of workers at the tin smelter. The study identified areas with the highest radiation hotspots, which workers should be aware of, based on the estimated annual effective dose calculated from the highest values. The work area with the highest estimated annual effective dose was identified at 1.30–2.01 mSv in both smelters. However, this was still below the IAEA-recommended limit for workers, except for slag storage at smelter B. Slag storage at smelter B requires special attention from workers and regulators, as this slag sample contains significant NORM, particularly 232th decay chain, which is the most significant contributor to external gamma exposure. The results of this assessment were used to optimize worker protection strategies, ensuring worker exposure remains as low as possible. Therefore, worker protection in the NORM industry should focus on source control through material management and ventilation, supported by regular radiation monitoring, appropriate work arrangements, the use of PPE, and consistent application of the ALARA principle.

## Data Availability

The original contributions presented in the study are included in the article/Supplementary material, further inquiries can be directed to the corresponding authors.
